# Documentation-derived nursing process indicators and in-hospital outcomes in patients with acute myocardial infarction undergoing PCI: A cohort study

**DOI:** 10.1097/MD.0000000000049375

**Published:** 2026-06-19

**Authors:** Haixia Ma, He Yin, Chunyan Wu, Jie Gao, Xiumei Yue, Hongbo Yu

**Affiliations:** a Catheterization Room, Beijing University of Chinese Medicine Dongfang Hospital Qinhuangdao Hospital (Qinhuangdao Hospital of Traditional Chinese Medicine), Qinhuangdao, Hebei Province, China; b The Nursing Department, Beijing University of Chinese Medicine Dongfang Hospital Qinhuangdao Hospital (Qinhuangdao Hospital of Traditional Chinese Medicine), Qinhuangdao, Hebei Province, China; c Cardiovascular Department 1, Beijing University of Chinese Medicine Dongfang Hospital Qinhuangdao Hospital (Qinhuangdao Hospital of Traditional Chinese Medicine), Qinhuangdao, Hebei Province, China; d Cardiovascular Department 2, Beijing University of Chinese Medicine Dongfang Hospital Qinhuangdao Hospital (Qinhuangdao Hospital of Traditional Chinese Medicine), Qinhuangdao, Hebei Province, China.

**Keywords:** acute myocardial infarction, in-hospital adverse outcomes, nursing documentation, percutaneous coronary intervention, process-of-care, risk stratification

## Abstract

In-hospital outcomes after acute myocardial infarction (AMI) treated with percutaneous coronary intervention remain variable. Documentation-derived nursing process indicators may act as proxy markers of clinical acuity, surveillance intensity, and care complexity, but their association with short-term outcomes remains unclear. This single-center retrospective cohort study included consecutive adults with AMI. Nursing process indicators were extracted from routine nursing records, including assessment frequency, monitoring intensity, documentation density, and selected observational items. The primary endpoint was a composite of all-cause in-hospital death, recurrent myocardial infarction, malignant arrhythmia, cardiogenic shock, major bleeding, or prolonged hospitalization. Multivariable logistic regression was used to estimate adjusted odds ratios (ORs). Intensive care unit admission was examined as a contextual care-setting variable but was not included in the final extended discrimination model. Internal model discrimination was assessed using receiver operating characteristic analysis and area under the curve comparison by DeLong test; no external validation was performed. Of 512 screened patients, 438 were included in the final analysis; the mean age was 61.4 years, and 73.5% were male. During hospitalization, 168 patients (38.4%) experienced the composite primary endpoint; all-cause in-hospital mortality occurred in 4.1%, and major adverse cardiovascular events occurred in 14.2%. After multivariable adjustment, higher nursing documentation density was associated with the composite endpoint (per additional record per day: adjusted OR: 1.29, 95% confidence interval [CI]: 1.08–1.55; *P* = .005), as was documented cardiac rhythm monitoring (adjusted OR: 1.88, 95% CI: 1.25–2.84; *P* = .002). In an internal discrimination analysis without external validation, the baseline clinical model showed an AUC of 0.71 (95% CI: 0.66–0.76), whereas the extended model including selected documentation-derived nursing process indicators showed an AUC of 0.76 (95% CI: 0.71–0.81; ΔAUC = 0.05; DeLong *P* = .012). In this real-world cohort of patients with AMI undergoing percutaneous coronary intervention, documentation-derived nursing process indicators were associated with in-hospital adverse outcomes and provided modest incremental internal discriminative information beyond traditional clinical predictors.

## 1. Introduction

Acute myocardial infarction (AMI) remains a major cause of in-hospital morbidity and mortality worldwide.^[1–3]^ Despite substantial advances in reperfusion strategies, including timely percutaneous coronary intervention (PCI), adverse in-hospital outcomes such as death, malignant arrhythmias, bleeding events, and cardiogenic shock continue to occur in a considerable proportion of patients.^[4–6]^ Risk stratification in patients with AMI undergoing PCI has traditionally relied on validated clinical risk models, such as the global registry of acute coronary events (GRACE) and thrombolysis in myocardial infarction (TIMI) risk scores 36 to 38, as well as demographic characteristics, markers of disease severity, laboratory parameters, and procedural factors, including Killip classification, left ventricular ejection fraction (LVEF), renal function, coronary disease burden, and coronary flow status.^[7–9]^ These tools and variables provide essential prognostic information, but they primarily capture baseline or procedure-related risk. In real-world clinical practice, substantial heterogeneity in in-hospital outcomes persists among patients with apparently similar clinical risk profiles.^[10]^ This observation suggests that conventional risk assessment frameworks, while essential, may not fully capture dynamic information generated during hospitalization, including changes in surveillance intensity, care complexity, and evolving clinical instability.^[11–13]^

Within the periprocedural management of AMI, nursing care is an integral component of routine clinical practice.^[14–16]^ Nursing activities include continuous monitoring, symptom assessment, early recognition of clinical deterioration, and documentation of potential complications throughout hospitalization.^[17–19]^ In this context, routinely recorded nursing documentation may provide information on how patients are observed and monitored over time. Importantly, documentation-derived nursing process indicators differ from intervention-based nursing models. They do not represent standardized nursing protocols, therapeutic interventions, or care bundles intended to modify outcomes. Rather, they are process-oriented indicators extracted from routine records, including assessment frequency, monitoring intensity, documentation density, and selected observational items. These indicators may reflect surveillance intensity and care complexity during hospitalization, but they should be interpreted as proxy markers rather than direct measures of nursing effectiveness or care quality.^[20–22]^

A key methodological consideration is that documentation-derived nursing process indicators are vulnerable to reverse causation and confounding by indication. Patients with more severe disease, evolving instability, or anticipated complications are more likely to receive closer monitoring and to generate more nursing documentation. Conversely, emerging complications may prompt more frequent assessments and additional records. Therefore, higher nursing documentation density may be associated with adverse outcomes because it captures real-time clinical concern, higher acuity, and greater care complexity, rather than because documentation itself causes adverse events.^[23–26]^ This distinction is essential when interpreting associations between nursing process indicators and in-hospital outcomes in observational data.

Previous studies examining outcomes in patients with AMI undergoing PCI have largely focused on medical risk factors, procedural and imaging characteristics, and pharmacological treatment strategies.^[27–29]^ These approaches have substantially advanced prognostic assessment and informed clinical decision-making.^[17,30]^ In contrast, nursing-related factors have often been incorporated in a limited or indirect manner.^[15,16,31]^ When considered, they are commonly represented by coarse indicators such as intensive care unit (ICU) admission status, levels of care, or nurse-to-patient ratios, which may insufficiently capture the complexity and dynamics of nursing care during hospitalization.^[32,33]^ As a result, relatively few investigations have systematically evaluated process-oriented nursing care characteristics, such as the frequency of documented assessments, monitoring intensity, and the breadth of nursing observations, or examined their statistical associations with in-hospital adverse outcomes.^[19,34,35]^ This gap in the literature highlights an underexplored dimension of inpatient care that may contribute to the observed heterogeneity in short-term outcomes among patients with similar clinical risk profiles.

Therefore, this study was designed as a descriptive and prognostic-associational analysis rather than a causal evaluation of nursing interventions or a quality-of-care assessment. The objectives were to characterize the distribution of documentation-derived periprocedural nursing process indicators, evaluate their associations with in-hospital adverse outcomes, and explore whether selected nursing documentation indicators provide incremental internal discriminative information beyond available conventional clinical and PCI-related variables.

## 2. Methods

### 2.1. Study design and setting

This was a single-center retrospective observational cohort study based on routinely collected clinical and nursing records. All consecutive adult patients with AMI who underwent PCI at Qinhuangdao Hospital of Traditional Chinese Medicine between January 2021 and December 2023 were screened for eligibility. No research-driven assignment, modification, or standardization of nursing care was performed.

The study was conducted at Qinhuangdao Hospital of Traditional Chinese Medicine, a tertiary referral center providing comprehensive cardiovascular care. The hospital has an established chest pain and AMI care pathway, 24-hour catheterization laboratory services, coronary care units, ICU availability, and a structured electronic nursing documentation system. During the study period, the hospital performed approximately 1000 PCI procedures annually, including approximately 300 PCI procedures for AMI.

Index PCI was defined as PCI performed for the acute infarct-related event during the index hospitalization, including primary PCI for ST-segment elevation myocardial infarction (STEMI), rescue PCI when applicable, and urgent or early invasive PCI for non-ST-segment elevation myocardial infarction (NSTEMI). Elective or staged non-culprit PCI performed after clinical stabilization was recorded when available but was not used to define the index PCI exposure window.

The observation period extended from hospital admission to discharge, during which all in-hospital outcomes were ascertained. Electronic timestamps were used to determine the timing of nursing documentation, PCI, ICU/coronary care unit (CCU)-level care, and outcome occurrence. For the primary analysis, documentation-derived nursing process indicators were calculated during the 1st 48 hours after admission or until the 1st adverse outcome, discharge, or death, whichever occurred 1st. Nursing documentation recorded after the 1st adverse outcome was excluded from the primary exposure calculation to reduce reverse causation and immortal time bias. Whole-hospitalization nursing indicators were retained for descriptive and sensitivity analyses.

### 2.2. Study population

**Figure 1.**
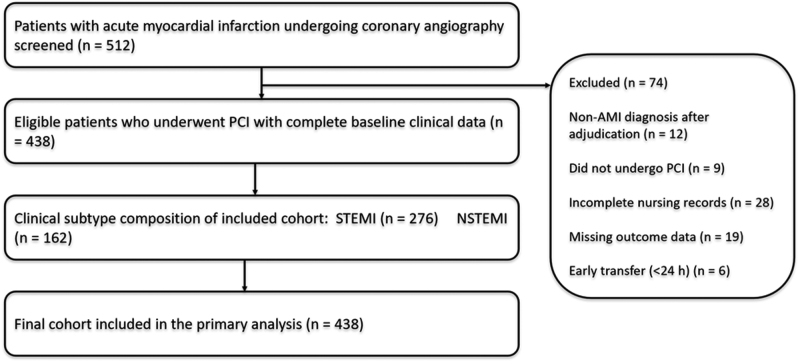
Participant flow diagram. The participant flow diagram shows screening, exclusions, and final inclusion in the analytic cohort. A total of 512 patients with suspected acute myocardial infarction who underwent coronary angiography were screened. Seventy-four patients were excluded: non-AMI diagnosis after adjudication, n = 21; no PCI during the index hospitalization, n = 15; incomplete or non-extractable nursing documentation, n = 18; missing in-hospital outcome data, n = 7; inter-hospital transfer or premature withdrawal from care, n = 9; and severe noncardiac comorbid conditions, n = 4. The final analytic cohort included 438 patients with AMI who underwent PCI. AMI = acute myocardial infarction, NSTEMI = non-ST-segment elevation myocardial infarction, PCI = percutaneous coronary intervention, STEMI = ST-segment elevation myocardial infarction.

The study population consisted of adult patients with AMI who underwent coronary angiography and received PCI during the study period. All consecutive eligible AMI-PCI patients during the study period were screened. A total of 512 patients were initially screened, of whom 438 met the eligibility criteria and were included in the final analysis. The number of patients excluded for each reason is reported in Figure 1 and Table S1, Supplemental Digital Content 1.

AMI was diagnosed according to the Fourth Universal Definition of Myocardial Infarction, based on evidence of acute myocardial injury with a rise and/or fall in cardiac troponin values and at least 1 feature of myocardial ischemia, including ischemic symptoms, new ischemic electrocardiographic changes, development of pathological *Q* waves, imaging evidence of new loss of viable myocardium or new regional wall motion abnormality, or identification of coronary thrombus by angiography. STEMI was defined as AMI with new ST-segment elevation in at least two contiguous leads or new/presumed new left bundle branch block in the appropriate clinical context. NSTEMI was defined as AMI without persistent ST-segment elevation but with elevated cardiac troponin and clinical evidence of myocardial ischemia.

If more than 1 eligible AMI hospitalization occurred for the same patient during the study period, only the 1st eligible hospitalization was included. Patients with prior PCI or prior coronary artery bypass grafting were not excluded if they met the diagnostic criteria for AMI and underwent PCI for the index event. Patients presenting with cardiogenic shock or resuscitated out-of-hospital cardiac arrest were eligible if PCI was performed and sufficient preevent nursing documentation was available.

The study protocol was reviewed and approved by the institutional ethics committee of Qinhuangdao Hospital of Traditional Chinese Medicine. Because this was a retrospective record-based study using anonymized data and involving no direct patient contact or intervention, the requirement for written informed consent was waived by the ethics committee. No patients declined participation because individual informed consent was not required for this anonymized retrospective record review. The study was conducted in accordance with the principles of the Declaration of Helsinki, and patient confidentiality was strictly maintained throughout the study.

### 2.3. Inclusion and exclusion criteria

Patients were eligible for inclusion if they met all of the following criteria: age ≥ 18 years at admission; diagnosis of AMI, including STEMI or NSTEMI, according to the criteria described above; receipt of PCI for the index AMI event during the index hospitalization; and availability of complete and extractable clinical and nursing documentation for the predefined exposure window.

Patients were excluded if any of the following conditions were present: non-AMI diagnosis after adjudication; no PCI performed during the index hospitalization; incomplete or non-extractable nursing documentation precluding reliable exposure assessment; missing in-hospital outcome data; inter-hospital transfer or premature withdrawal from care before definitive outcome ascertainment; or severe noncardiac comorbid conditions expected to dominate short-term prognosis.

Severe noncardiac comorbid conditions were operationally defined as advanced malignancy with expected survival <6 months, end-stage liver disease, end-stage noncardiac systemic disease, severe active infection or sepsis present at admission, or other documented terminal conditions judged by the treating team to dominate short-term prognosis independently of AMI. Inter-hospital transfer was an exclusion criterion because subsequent outcomes and nursing documentation after transfer could not be reliably ascertained. Early in-hospital death with insufficient nursing documentation was quantified separately because exclusion of such patients may introduce selection bias.

### 2.4. Data sources and data collection

Data were obtained from routinely maintained hospital information systems. Demographic characteristics, comorbid conditions, laboratory results, medication use, echocardiographic parameters, and hospitalization details were extracted from the electronic health record system. Procedural and angiographic variables related to PCI, including the number of diseased vessels, infarct-related artery (IRA), pre-PCI TIMI flow, and procedural timing, were obtained from the institutional PCI registry database where available and cross-validated against catheterization laboratory records.

Detailed information on documentation-derived nursing process indicators, including nursing assessments, vital sign monitoring, clinical observations, and documentation frequency, was retrieved from the institutional electronic nursing documentation system. Nursing process variables were extracted using a mixed approach. Structured fields were exported electronically from the nursing information system, whereas semi-structured or free-text entries were manually reviewed according to prespecified coding rules. The coding rules used to extract nursing documentation variables are provided in Table S3, Supplemental Digital Content 2.

All variables were derived from preexisting clinical and nursing documentation without research-driven modification or Supplementary Data, Supplemental Digital Content 1 collection. Data extraction was performed by trained investigators using standardized extraction forms. A random sample of 120 records was independently reviewed to validate the extraction of nursing documentation variables. Percent agreement and Cohen kappa were used to assess extraction consistency for binary indicators, and the validation results are provided in Table S4, Supplemental Digital Content 3. Any discrepancies identified during the verification process were resolved through discussion and consensus.

Missing data were evaluated for each variable before statistical analysis. Variables with >20% missing data were prespecified for exclusion from multivariable models. After application of the eligibility criteria, variables included in the primary analysis had no missing values; therefore, no imputation was required for the primary models. Missing data for all analyzed variables are reported in Table S5, Supplemental Digital Content 4.

### 2.5. Definition of documentation-derived nursing process indicators

For this study, nursing variables were defined as documentation-derived nursing process **indicators** rather than direct nursing interventions. They were used to characterize recorded patterns of patient observation, monitoring, and clinical documentation and were not interpreted as measures of nursing quality, nurse performance, or causal treatment exposure.

For the primary analysis, the nursing exposure window was defined as the 1st 48 hours after admission or the period before the 1st adverse outcome, discharge, or death, whichever occurred 1st. Documentation recorded after the 1st adverse outcome was excluded from exposure calculation. Whole-hospitalization nursing documentation measures were used only for descriptive and sensitivity analyses.

Total nursing assessment records were defined as structured clinical nursing assessment entries documented in the electronic nursing system, including admission nursing assessment, shift assessment, pre-PCI assessment, post-PCI assessment, symptom-focused assessment, and complication-related nursing observation records. Administrative entries, billing records, order acknowledgements, duplicate system-generated records, and medication administration records were not counted.

Vital sign monitoring records included documented measurements of systolic and diastolic blood pressure, heart rate, respiratory rate, oxygen saturation, and body temperature. Vital sign monitoring frequency was calculated as the number of eligible vital sign monitoring records per exposure day.

Nursing documentation density was defined as the number of eligible nursing-related clinical entries per exposure day within the predefined exposure window. Eligible entries included structured nursing assessments, vital sign monitoring records, pain assessment records, access-site or bleeding observation records, cardiac rhythm monitoring observations, fluid balance records, and complication-related nursing observations. Administrative entries, billing items, medication administration records, order acknowledgements, duplicate entries, and automatically generated nonclinical system logs were excluded.

Content-related nursing indicators were treated as binary variables according to whether the corresponding observation was documented during the predefined exposure window. These indicators included pain assessment, bleeding observation, access-site inspection, cardiac rhythm monitoring, and fluid balance monitoring. Binary nursing indicators were interpreted as indicators of recorded documentation rather than direct confirmation of care delivery. Documented cardiac rhythm monitoring was interpreted as evidence of rhythm surveillance or telemetry-related documentation, which may reflect physician indication, institutional monitoring pathways, or patient acuity rather than nurse-initiated care intensity. Bleeding observation documentation may reflect bleeding risk, access-site concerns, antithrombotic exposure, or active bleeding. Fluid balance monitoring may reflect heart failure, renal dysfunction, hemodynamic instability, shock, or ICU/CCU-level care.

ICU/CCU-level care was defined as any intensive or coronary care unit-level care during hospitalization, including direct ICU/CCU admission at presentation or immediately after PCI and subsequent ICU transfer after initial non-ICU management. ICU/CCU-level care was treated as a contextual care-setting and severity-related variable, not as a documentation-derived nursing process indicator. ICU transfer after initial non-ICU management was analyzed separately as a secondary outcome.

### 2.6. Outcome measures

The primary outcome was a composite endpoint defined as the occurrence of at least 1 severe in-hospital adverse outcome during the index hospitalization. The primary composite endpoint consisted of any of the following individual events: all-cause in-hospital death, recurrent myocardial infarction, malignant arrhythmia, cardiogenic shock, or major bleeding. Each patient was counted once for the primary composite endpoint regardless of the number of component events.

Secondary outcomes included the individual components of the primary composite endpoint, major adverse cardiovascular events (MACE), ICU transfer after initial non-ICU management, and prolonged length of stay. MACE was defined as all-cause death, recurrent myocardial infarction, malignant arrhythmia, or cardiogenic shock during hospitalization and was reported as a secondary composite outcome to avoid double-counting within the primary composite endpoint. Prolonged length of stay was defined as hospitalization longer than 7 days and was analyzed as an exploratory secondary outcome because it may be influenced by complications, care processes, and documentation volume.

Major bleeding was defined as Bleeding Academic Research Consortium type 3 or 5 bleeding during hospitalization. Malignant arrhythmia was defined as sustained ventricular tachycardia, ventricular fibrillation, high-grade atrioventricular block requiring treatment or temporary pacing, asystole, or other life-threatening rhythm disturbances requiring urgent intervention. Recurrent myocardial infarction was defined according to recurrent ischemic symptoms or electrocardiographic changes with a new rise in cardiac biomarkers after the index event. Cardiogenic shock was defined as persistent hypotension with evidence of end-organ hypoperfusion requiring pharmacologic or mechanical circulatory support.

All outcomes were ascertained using data from the electronic health record system, including discharge diagnoses, daily progress notes, laboratory findings, procedural records, and nursing documentation. Outcome information was cross-validated across multiple sources. Outcome adjudication was performed independently by 2 investigators who were blinded to documentation-derived nursing process indicators and analytical hypotheses. In cases of disagreement, outcomes were reviewed jointly and resolved by consensus, with adjudication by a 3rd investigator when necessary. The time from admission to the 1st adverse outcome was extracted when available.

### 2.7. Covariates

Baseline demographic and clinical variables were included as covariates to account for differences in patient characteristics and disease severity. These variables included age, sex, Killip class, LVEF, hypertension, diabetes mellitus, prior myocardial infarction, and serum creatinine.

Procedural and angiographic variables related to coronary anatomy and PCI were included to reflect coronary disease burden and procedural complexity. These variables included the number of diseased coronary vessels, IRA, and pre-PCI TIMI flow. Admission systolic blood pressure and admission heart rate were additionally considered in sensitivity analyses where appropriate.

Peak troponin I was summarized descriptively but was not included in the primary adjustment model because peak values may occur after PCI or after early adverse events and may therefore represent a post-baseline or post-deterioration variable. Peak troponin I was considered in sensitivity analyses where appropriate.

Key in-hospital medications included antiplatelet agents, anticoagulants, statins, angiotensin-converting enzyme inhibitors, angiotensin receptor blockers or angiotensin receptor–neprilysin inhibitors, and β-blockers. Medication variables were summarized descriptively but were not included in the primary adjustment model because in-hospital medication use may occur after clinical deterioration and could introduce post-baseline adjustment bias. Medication variables were considered only in sensitivity analyses when timing information was available.

Established AMI risk scores, such as GRACE and TIMI, were considered conceptually. However, complete variables required for formal GRACE or TIMI score calculation were not uniformly available in the retrospective dataset. Therefore, the primary reference model was constructed using routinely available demographic, clinical severity, comorbidity, renal function, and PCI-related variables. The purpose of the model was not to replace validated AMI risk scores but to evaluate whether nursing documentation indicators provided incremental internal discriminative information.

### 2.8. Statistical analysis

Baseline characteristics, nursing documentation indicators, and clinical outcomes were summarized using descriptive statistics. Continuous variables were expressed as mean ± standard deviation when approximately normally distributed or as median with interquartile range (IQR) when skewed. Categorical variables were presented as counts and percentages. Continuous variables were compared using Student *t* test or the Mann–Whitney *U* test, as appropriate. Categorical variables were compared using the χ^2^ test or Fisher exact test, depending on cell counts.

Baseline characteristics were primarily compared between patients with and without the primary composite endpoint. Comparisons between STEMI and NSTEMI patients were retained as supplementary descriptive analyses. Documentation-derived nursing process indicators were compared according to primary composite endpoint status and were also described by clinical severity and care setting in supplementary analyses.

The primary association analyses were conducted using logistic regression models, with the primary composite endpoint as the dependent variable. In univariate analyses, each documentation-derived nursing process indicator was examined separately to estimate odds ratios (ORs) with 95% confidence intervals (CIs). In multivariable analyses, each nursing process indicator was modeled separately after adjustment for the prespecified clinical covariates to reduce collinearity among nursing documentation variables. The primary adjustment model included age, sex, Killip class, hypertension, diabetes mellitus, prior myocardial infarction, LVEF, serum creatinine, number of diseased vessels, IRA, and pre-PCI TIMI flow.

ICU/CCU-level care was evaluated separately as a contextual care-setting variable and was not included in the final extended discrimination model. The final extended model included the clinical model plus selected nursing documentation indicators, specifically nursing documentation density and documented cardiac rhythm monitoring.

Variable selection for multivariable modeling was based on clinical relevance, prior evidence, temporal availability, and events-per-variable considerations rather than univariate *P* values alone. The number of primary outcome events was used to assess potential overfitting. Continuous predictors were assessed for linearity in the logit using visual inspection and restricted cubic spline analyses where appropriate.

Multicollinearity among nursing-related variables and ICU/CCU-level care was assessed using correlation matrices and variance inflation factors (VIFs). Correlation coefficients and VIFs are reported in Table S6, Supplemental Digital Content 5.

Model discrimination was evaluated using receiver operating characteristic curves and the area under the curve (AUC). AUCs were compared using DeLong test, and changes in discrimination were quantified using ΔAUC. Model calibration was assessed using Brier score, calibration intercept, calibration slope, and the Hosmer–Lemeshow goodness-of-fit test. Internal validation was performed using bootstrap resampling to estimate optimism-corrected discrimination. Detailed model performance metrics are reported in Table S10, Supplemental Digital Content 6. These analyses were conducted to assess internal discrimination and calibration rather than to develop or validate a clinical prediction tool. No external validation was performed.

Prespecified subgroup analyses were performed to examine the consistency of the association between nursing documentation density and the primary composite endpoint across clinically relevant subgroups defined by age, sex, AMI subtype, and care setting. Interaction terms were included to assess potential effect modification, and P values for interaction were reported. Subgroup-specific sample sizes and event counts are reported in Table S7, Supplemental Digital Content 7, and subgroup analyses were considered exploratory.

Several sensitivity analyses were conducted to evaluate robustness. These included alternative exposure windows, including the 1st 24 hours after admission and the 1st 24 hours after PCI; use of whole-hospitalization documentation density; exclusion of patients with in-hospital death; restriction to patients without ICU/CCU-level care; alternative outcome definitions; additional adjustment for admission systolic blood pressure and heart rate; and complete-case analysis. Sensitivity analyses are reported in Table S8, Supplemental Digital Content 8.

All statistical analyses were performed using Stata. All tests were 2-sided, and a *P* value of <.05 was considered statistically significant. No formal adjustment for multiplicity was applied; therefore, secondary outcome analyses, subgroup analyses, and sensitivity analyses were interpreted as exploratory, with emphasis placed on consistency of direction and magnitude rather than isolated *P* values.

## 3. Results

### 3.1. Patient selection

A total of 512 patients with suspected acute myocardial infarction who underwent coronary angiography between January 2021 and December 2023 were initially screened. After adjudication, 74 patients were excluded for the following reasons: non-AMI diagnosis, n = 21; no PCI during the index hospitalization, n = 15; incomplete or non-extractable nursing documentation, n = 18; missing in-hospital outcome data, n = 7; inter-hospital transfer or premature withdrawal from care, n = 9; and severe noncardiac comorbid conditions expected to dominate short-term prognosis, n = 4. Among the 18 patients excluded because of incomplete nursing documentation, 4 died early before sufficient nursing records were available for reliable exposure assessment. Finally, 438 patients with AMI who underwent PCI were included in the analytic cohort (Figure 1 and Table S1, Supplemental Digital Content 1).

### 3.2. Baseline clinical characteristics

Baseline clinical characteristics according to the occurrence of the primary composite endpoint are presented in Table 1. Among the 438 included patients, the mean age was 61.42 ± 11.36 years, and 322 patients (73.52%) were male. During hospitalization, 168 patients experienced the primary composite endpoint, whereas 270 did not.

**Table 1:** Baseline clinical characteristics of the study population according to AMI subtype.

Characteristic	Missing, n (%)	Total (n = 438)	STEMI (n = 276)	NSTEMI (n = 162)	*P* value
Age (yr)	0 (0.0)	61.42 ± 11.36	60.18 ± 11.02	63.48 ± 11.71	.004
Male sex, n (%)	0 (0.0)	322 (73.52)	215 (77.90)	107 (66.05)	.006
Body mass index, kg/m^2^	0 (0.0)	25.18 ± 3.41	25.26 ± 3.36	25.05 ± 3.49	.521
Killip class ≥ II, n (%)	0 (0.0)	126 (28.77)	96 (34.78)	30 (18.52)	<.001
Left ventricular ejection fraction, %	0 (0.0)	52.36 ± 8.94	50.92 ± 8.76	54.82 ± 8.71	<.001
Hypertension, n (%)	0 (0.0)	246 (56.16)	149 (53.99)	97 (59.88)	.232
Diabetes mellitus, n (%)	0 (0.0)	158 (36.07)	90 (32.61)	68 (41.98)	.047
Prior myocardial infarction, n (%)	0 (0.0)	52 (11.87)	27 (9.78)	25 (15.43)	.071
Current smoking, n (%)	0 (0.0)	201 (45.89)	139 (50.36)	62 (38.27)	.014
Serum creatinine (μmol/L)	0 (0.0)	86.74 ± 21.83	84.92 ± 20.77	89.86 ± 23.24	.028
Peak troponin I (ng/mL)	0 (0.0)	14.60 (7.20–27.80)	23.50 (14.10–34.20)	7.10 (3.80–11.90)	<.001
Number of diseased vessels ≥ 2, n (%)	0 (0.0)	231 (52.74)	132 (47.83)	99 (61.11)	.008
Infarct-related artery, n (%)	0 (0.0)				.012
└ Left anterior descending artery	0 (0.0)	196 (44.75)	138 (50.00)	58 (35.80)	
└ Right coronary artery	0 (0.0)	154 (35.16)	92 (33.33)	62 (38.27)	
└ Left circumflex artery	0 (0.0)	88 (20.09)	46 (16.67)	42 (25.93)	
Pre-PCI TIMI flow ≤ 1, n (%)	0 (0.0)	214 (48.86)	173 (62.68)	41 (25.31)	<.001

Values are presented as mean ± standard deviation, median (interquartile range), or number (percentage), as appropriate. *P* values compare STEMI and NSTEMI groups. Continuous variables were compared using Student *t* test or the Mann–Whitney *U* test, as appropriate. Categorical variables were compared using the χ^2^ test or Fisher exact test, as appropriate.

AMI = acute myocardial infarction, NSTEMI = non-ST-segment elevation myocardial infarction, PCI = percutaneous coronary intervention, STEMI = ST-segment elevation myocardial infarction, TIMI = thrombolysis in myocardial infarction.

Compared with patients without the primary composite endpoint, those with the endpoint were older, more frequently had Killip class ≥ II, had lower LVEF, higher serum creatinine, more frequent multivessel coronary artery disease, and a higher proportion of pre-PCI TIMI flow ≤ 1. Admission systolic blood pressure was lower and admission heart rate was higher among patients with the primary composite endpoint. Peak troponin I showed a skewed distribution and was therefore reported as median with IQR.

Baseline characteristics according to AMI subtype are shown in Table S2, Supplemental Digital Content 9. Patients with NSTEMI were older than those with STEMI, whereas male sex was more common in the STEMI group. Patients with STEMI more frequently presented with Killip class ≥ II, had lower LVEF, higher peak troponin I levels, and more frequent pre-PCI TIMI flow ≤ 1. In contrast, diabetes mellitus and multivessel coronary artery disease were more common among patients with NSTEMI. Missing data for variables included in the analysis are summarized in Table S5, Supplemental Digital Content 4; after application of the eligibility criteria, variables included in the primary analysis had no missing values.

### 3.3. Documentation-derived nursing process indicators

Documentation-derived nursing process indicators according to primary composite endpoint status are summarized in Table 2. For the primary analysis, nursing records were counted during the 1st 48 hours after admission or until the 1st adverse outcome, discharge, or death, whichever occurred 1st. Documentation recorded after the 1st adverse outcome was excluded from the primary exposure calculation.

**Table 2:** Documentation-derived nursing process indicators according to ICU/CCU-level care status.

Nursing process indicator	Overall (n = 438)	Any ICU/CCU-level care (n = 124)	No ICU/CCU-level care (n = 314)	*P* value
Total nursing assessment records, n	18.42 ± 6.73	26.58 ± 7.94	15.09 ± 4.82	<.001
Vital sign monitoring records per day, n	9.36 ± 3.21	13.74 ± 3.89	7.62 ± 2.41	<.001
Pain assessment documented, n (%)	371 (84.70)	116 (93.55)	255 (81.21)	.002
Bleeding observation documented, n (%)	328 (74.89)	109 (87.90)	219 (69.75)	<.001
Access-site inspection documented, n (%)	342 (78.08)	112 (90.32)	230 (73.25)	<.001
Cardiac rhythm monitoring documented, n (%)	286 (65.30)	118 (95.16)	168 (53.50)	<.001
Fluid balance monitoring documented, n (%)	301 (68.72)	114 (91.94)	187 (59.55)	<.001
Nursing documentation density, records/day	4.62 ± 1.58	6.38 ± 1.71	3.93 ± 1.12	<.001

Values are presented as mean ± standard deviation or number (percentage), as appropriate. ICU/CCU-level care was defined as any intensive or coronary care unit-level care during hospitalization, including direct ICU/CCU admission at presentation or immediately after PCI and subsequent ICU transfer after initial non-ICU management. For the primary analysis, nursing records were counted during the 1st 48 hours after admission or until the 1st adverse outcome, discharge, or death, whichever occurred 1st; documentation recorded after the 1st adverse outcome was excluded. *P* values compare patients with and without ICU/CCU-level care.

CCU = coronary care unit, ICU = intensive care unit.

Compared with patients without the primary composite endpoint, patients with the endpoint had more total nursing assessment records (22.51 ± 7.42 vs 15.88 ± 4.93), higher vital sign monitoring frequency (11.08 ± 3.42 vs 8.29 ± 2.61 records/day), and greater nursing documentation density (5.59 ± 1.69 vs 4.02 ± 1.15 records/day). Documentation of selected nursing observations was also more frequent among patients with the primary composite endpoint, including pain assessment (91.67% vs 80.37%), bleeding observation (85.71% vs 68.15%), access-site inspection (86.90% vs 72.59%), cardiac rhythm monitoring (79.17% vs 56.67%), and fluid balance monitoring (81.55% vs 60.74%).

The coding rules for documentation-derived nursing process indicators are provided in Table S3, Supplemental Digital Content 2. Validation of the extraction procedure is shown in Table S4, Supplemental Digital Content 3. In the validation sample, percent agreement ranged from 95.0% to 96.7% for binary nursing documentation indicators, and Cohen kappa values ranged from 0.84 to 0.93, indicating high consistency between extracted variables and manual review.

Documentation-derived nursing indicators also varied according to clinical severity and care setting (Table S9, Supplemental Digital Content 10). Patients with Killip class III to IV had more total nursing assessment records, higher vital sign monitoring frequency, and greater nursing documentation density than those with Killip class I to II. Similar patterns were observed among patients with periprocedural complications and among those receiving ICU/CCU-level care.

A total of 124 patients received ICU/CCU-level care at some point during hospitalization. This group included 52 patients directly admitted to ICU/CCU-level care at presentation or immediately after PCI and 72 patients transferred to ICU/CCU-level care after initial non-ICU management. Therefore, ICU/CCU-level care and ICU transfer represented related but distinct variables. ICU/CCU-level care was treated as a contextual care-setting variable rather than as a documentation-derived nursing process indicator.

### 3.4. Incidence and components of in-hospital adverse outcomes

During hospitalization, 168 patients (38.36%) experienced the primary composite endpoint (Table 3). All-cause in-hospital mortality occurred in 18 patients (4.11%), and MACE occurred in 62 patients (14.16%). Major bleeding was observed in 39 patients (8.90%), severe cardiac arrhythmia in 54 patients (12.33%), ICU transfer after initial non-ICU management in 72 patients (16.44%), and prolonged length of stay in 129 patients (29.45%).

**Table 3:** Incidence of primary composite endpoint components and secondary outcomes.

Outcome	Overall (n = 438)	STEMI (n = 276)	NSTEMI (n = 162)	*P* value
Primary composite endpoint	168 (38.36)	121 (43.84)	47 (29.01)	.002
All-cause in-hospital mortality	18 (4.11)	15 (5.43)	3 (1.85)	.049
Recurrent myocardial infarction	14 (3.20)	10 (3.62)	4 (2.47)	.505
Malignant arrhythmia	54 (12.33)	41 (14.86)	13 (8.02)	.031
Cardiogenic shock	24 (5.48)	19 (6.88)	5 (3.09)	.087
Major bleeding	39 (8.90)	28 (10.14)	11 (6.79)	.217
Secondary outcomes				
MACE	62 (14.16)	46 (16.67)	16 (9.88)	.041
ICU transfer after initial non-ICU management	72 (16.44)	58 (21.01)	14 (8.64)	<.001
Prolonged length of stay > 7 days	129 (29.45)	89 (32.25)	40 (24.69)	.086
One adverse outcome component	91 (20.78)	64 (23.19)	27 (16.67)	.105
Two adverse outcome components	51 (11.64)	38 (13.77)	13 (8.02)	.071
Three or more adverse outcome components	26 (5.94)	19 (6.88)	7 (4.32)	.273

Values are presented as number (percentage). The primary composite endpoint was defined as the occurrence of at least one of the following events during hospitalization: all-cause death, recurrent myocardial infarction, malignant arrhythmia, cardiogenic shock, or major bleeding. Each patient was counted once for the primary composite endpoint, regardless of the number of component events. Recurrent myocardial infarction was defined according to recurrent ischemic symptoms or electrocardiographic changes with a new rise in cardiac biomarkers after the index event. Malignant arrhythmia was defined as sustained ventricular tachycardia, ventricular fibrillation, high-grade atrioventricular block requiring treatment or temporary pacing, asystole, or other life-threatening rhythm disturbances requiring urgent intervention. Major bleeding was defined as Bleeding Academic Research Consortium type 3 or 5 bleeding. MACE was defined as all-cause death, recurrent myocardial infarction, malignant arrhythmia, or cardiogenic shock. ICU transfer referred to transfer to ICU/CCU-level care after initial non-ICU management. Prolonged length of stay was analyzed as an exploratory secondary outcome. *P* values compare STEMI and NSTEMI groups.

AMI = acute myocardial infarction, CCU = coronary care unit, ICU = intensive care unit, MACE = major adverse cardiovascular events, NSTEMI = non-ST-segment elevation myocardial infarction, PCI = percutaneous coronary intervention, STEMI = ST-segment elevation myocardial infarction, TIMI = thrombolysis in myocardial infarction.

Outcome rates differed by AMI subtype. All-cause in-hospital mortality was higher among patients with STEMI than among those with NSTEMI (5.43% vs 1.85%), as was the incidence of MACE (16.67% vs 9.88%). Severe cardiac arrhythmia was also more frequent in the STEMI group (14.86% vs 8.02%). ICU transfer after initial non-ICU management occurred more commonly among patients with STEMI than among those with NSTEMI (21.01% vs 8.64%). The incidence of prolonged length of stay did not differ significantly between AMI subtypes.

Overlap among adverse outcome components was common. Among the 168 patients with the primary composite endpoint, 91 patients (54.17%) had 1 adverse outcome component, 51 patients (30.36%) had 2 components, and 26 patients (15.48%) had 3 or more components. The median time from admission to the 1st adverse event was 53 hours (IQR, 28–96 hours). Because only 18 in-hospital deaths occurred, mortality was reported descriptively and was not analyzed using a separate, fully adjusted multivariable model.

### 3.5. Association between documentation-derived nursing process indicators and the primary composite endpoint

Univariate and multivariable associations between documentation-derived nursing process indicators and the primary composite endpoint are summarized in Table 4. In univariate analyses, total nursing assessment records, vital sign monitoring frequency, nursing documentation density, bleeding observation documentation, cardiac rhythm monitoring documentation, fluid balance monitoring documentation, and ICU/CCU-level care were associated with the primary composite endpoint.

**Table 4:** Associations between documentation-derived nursing process indicators and the primary composite endpoint.

Nursing process indicator	Univariate OR (95% CI)	*P* value	Adjusted OR (95% CI)	*P* value
Total nursing assessment records, per 5-record increase	1.42 (1.25–1.61)	<.001	1.21 (1.05–1.40)	.009
Vital sign monitoring frequency, per 3 records/day increase	1.36 (1.20–1.55)	<.001	1.18 (1.03–1.36)	.018
Pain assessment documented	1.89 (1.22–2.93)	.004	1.32 (0.83–2.11)	.240
Bleeding observation documented	2.11 (1.45–3.07)	<.001	1.49 (1.01–2.21)	.045
Access-site inspection documented	1.96 (1.32–2.91)	.001	1.38 (0.91–2.08)	.128
Cardiac rhythm monitoring documented	2.84 (1.98–4.08)	<.001	1.88 (1.25–2.84)	.002
Fluid balance monitoring documented	2.47 (1.71–3.57)	<.001	1.61 (1.10–2.36)	.015
Nursing documentation density, per 1 record/day increase	1.58 (1.34–1.86)	<.001	1.29 (1.08–1.55)	.005
ICU/CCU-level care	3.92 (2.74–5.62)	<.001	2.47 (1.61–3.78)	<.001

ORs and 95% CIs were estimated using logistic regression models. The outcome was the primary composite endpoint. Each nursing process indicator was modeled separately after adjustment for age, sex, Killip class, hypertension, diabetes mellitus, prior myocardial infarction, left ventricular ejection fraction, serum creatinine, number of diseased vessels, infarct-related artery, and pre-PCI TIMI flow. ICU/CCU-level care was evaluated separately as a contextual care-setting variable and was not included in the final extended discrimination model.

CCU = coronary care unit, CI = confidence interval, ICU = intensive care unit, OR = odds ratio, PCI = percutaneous coronary intervention, TIMI= thrombolysis in myocardial infarction.

After adjustment for age, sex, Killip class, hypertension, diabetes mellitus, prior myocardial infarction, LVEF, serum creatinine, number of diseased vessels, IRA, and pre-PCI TIMI flow, several documentation-derived nursing process indicators remained associated with the primary composite endpoint. Higher nursing assessment volume was associated with increased odds of the primary composite endpoint (per 5-record increase: adjusted OR: 1.21, 95% CI: 1.05–1.40; *P* = .009). Vital sign monitoring frequency was also associated with the endpoint (per 3 records/day increase: adjusted OR: 1.18, 95% CI: 1.03–1.36; *P* = .018). Nursing documentation density remained associated with the primary composite endpoint (per 1 record/day increase: adjusted OR: 1.29, 95% CI: 1.08–1.55; *P* = .005).

Among binary documentation indicators, bleeding observation documentation (adjusted OR: 1.49, 95% CI: 1.01–2.21; *P* = .045), cardiac rhythm monitoring documentation (adjusted OR: 1.88, 95% CI: 1.25–2.84; *P* = .002), and fluid balance monitoring documentation (adjusted OR: 1.61, 95% CI: 1.10–2.36; *P* = .015) remained associated with the primary composite endpoint. Pain assessment documentation was not significant after multivariable adjustment (adjusted OR: 1.32, 95% CI: 0.83–2.11; *P* = .240). ICU/CCU-level care was strongly associated with the primary composite endpoint when evaluated separately as a contextual care-setting variable (adjusted OR: 2.47, 95% CI: 1.61–3.78; *P* < .001), but it was not included in the final extended discrimination model.

Conventional clinical predictors were also associated with the primary composite endpoint in the adjusted clinical model. Higher Killip class, lower LVEF, higher serum creatinine, multivessel coronary artery disease, and pre-PCI TIMI flow ≤ 1 were associated with increased odds of the primary composite endpoint. Correlation coefficients and VIFs for nursing-related variables are reported in Table S6, Supplemental Digital Content 5. No VIF exceeded 5, suggesting no severe multicollinearity.

### 3.6. Discrimination, calibration, and internal validation of models

**Figure 2.**
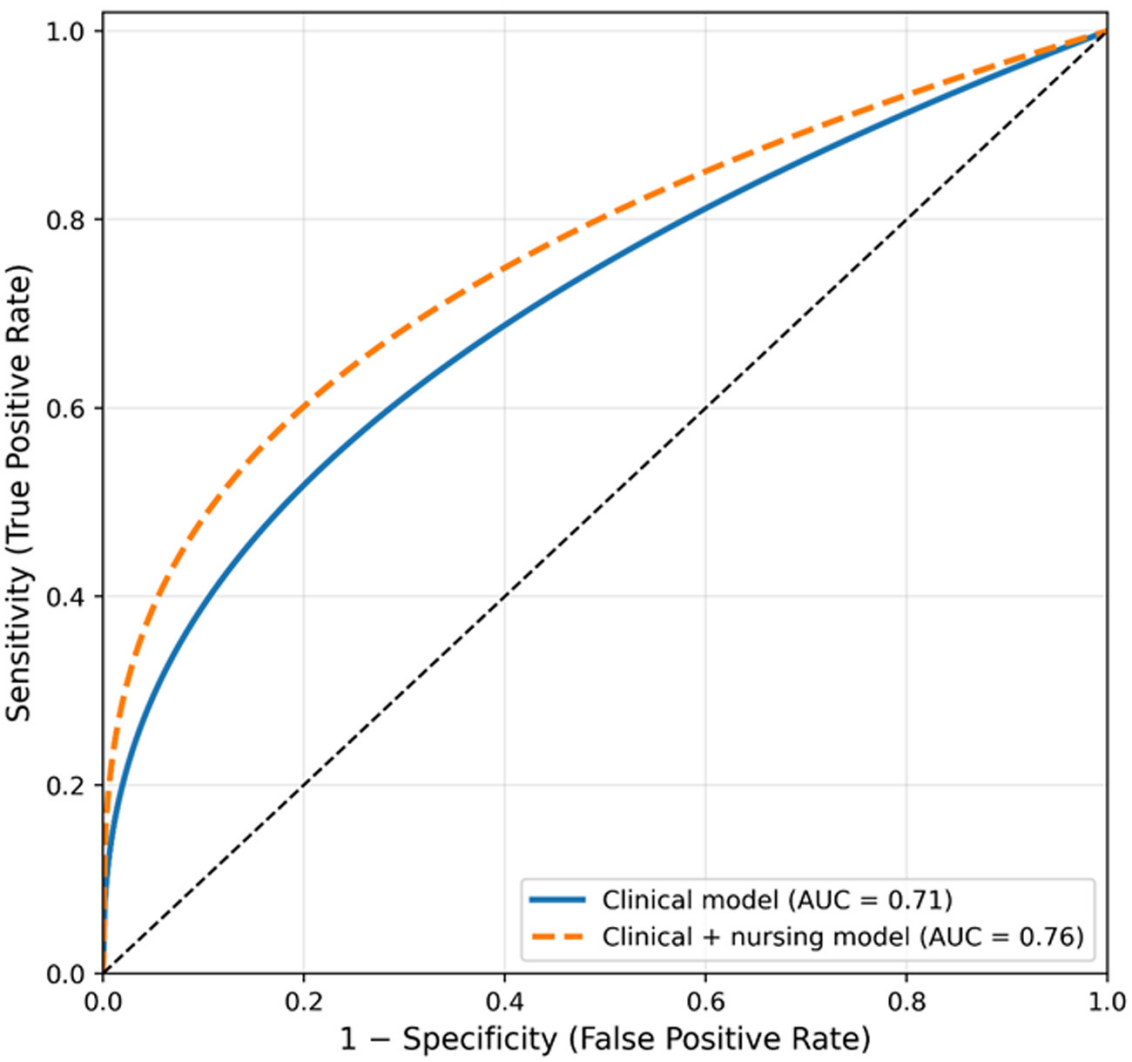
Receiver operating characteristic curves for the clinical and extended models. Receiver operating characteristic curves generated from patient-level predicted probabilities comparing the clinical model and the extended model for the primary composite endpoint. The clinical model included age, sex, Killip class, hypertension, diabetes mellitus, prior myocardial infarction, left ventricular ejection fraction, serum creatinine, number of diseased vessels, infarct-related artery, and pre-PCI TIMI flow. The extended model additionally included nursing documentation density and documented cardiac rhythm monitoring. The AUC was 0.71 (95% CI: 0.66–0.76) for the clinical model and 0.76 (95% CI: 0.71–0.81) for the extended model. The difference in AUC was statistically significant by DeLong test (ΔAUC = 0.05; *P* = .012). The curves were generated using patient-level model predictions rather than schematic smoothing. AUC = area under the curve, CI = confidence interval, PCI = percutaneous coronary intervention, TIMI = thrombolysis in myocardial infarction.

Model performance is summarized in Table 5 and Figure 2, withdetailed discrimination, calibration, and internal validation metrics provided in Table S10, Supplemental Digital Content 6. The baseline clinical model showed moderate internal discrimination for the primary composite endpoint, with an AUC of 0.71 (95% CI: 0.66–0.76). After adding selected documentation-derived nursing process indicators, including nursing documentation density and documented cardiac rhythm monitoring, the AUC increased to 0.76 (95% CI: 0.71–0.81), corresponding to a modest but statistically significant increase in internal discrimination (ΔAUC = 0.05; DeLong *P* = .012).

**Table 5:** Discrimination, calibration, and internal validation of models for the primary composite endpoint.

Model performance metric	Clinical model	Clinical + nursing documentation model
Apparent AUC (95% CI)	0.71 (0.66–0.76)	0.76 (0.71–0.81)
ΔAUC	Reference	0.05
DeLong *P* value	—	.012
Optimism-corrected AUC	0.70	0.75
Brier score	0.205	0.192
Calibration intercept	0.00	0.00
Calibration slope	0.94	0.96
Hosmer–Lemeshow *P* value	0.418	.536

The clinical model included age, sex, Killip class, hypertension, diabetes mellitus, prior myocardial infarction, left ventricular ejection fraction, serum creatinine, number of diseased vessels, infarct-related artery, and pre-PCI TIMI flow. The clinical + nursing documentation model additionally included nursing documentation density and documented cardiac rhythm monitoring. AUCs were compared using DeLong test. Internal validation was performed using bootstrap resampling. This analysis evaluated internal discrimination and calibration and was not intended to establish a validated clinical prediction tool.

AUC = area under the curve, CI = confidence interval, PCI = percutaneous coronary intervention, TIMI= thrombolysis in myocardial infarction.

Bootstrap internal validation showed an optimism-corrected AUC of 0.70 for the clinical model and 0.75 for the clinical plus nursing documentation model. Calibration assessment showed acceptable model calibration, with Brier scores of 0.205 and 0.192, calibration slopes of 0.94 and 0.96, and Hosmer–Lemeshow *P* values of .418 and .536 for the clinical and extended models, respectively.

### 3.7. Subgroup and sensitivity analyses

Subgroup analyses for nursing documentation density are presented in Table S7, Supplemental Digital Content 7. The association between nursing documentation density and the primary composite endpoint was generally consistent across age, sex, AMI subtype, and care-setting subgroups, with no statistically significant interaction observed. However, these analyses were exploratory because some subgroup-specific event counts were limited.

Sensitivity analyses are shown in Table S8, Supplemental Digital Content 8. In an analysis restricted to patients without ICU/CCU-level care, nursing documentation density remained associated with the primary composite endpoint (adjusted OR: 1.22 per additional record/day, 95% CI: 1.01–1.48; *P* = .041). After excluding patients with in-hospital death, the association also remained consistent (adjusted OR: 1.25 per additional record/day, 95% CI: 1.04–1.51; *P* = .018).

## 4. Discussion

Using real-world periprocedural clinical and nursing documentation data, this study evaluated the associations between documentation-derived nursing process indicators and in-hospital adverse outcomes among patients with acute myocardial infarction undergoing PCI. Several indicators reflecting documentation volume, monitoring frequency, and recorded observational coverage were associated with adverse in-hospital events. In addition, adding selected nursing documentation indicators to a conventional clinical model was associated with a modest increase in internal model discrimination. These findings should be interpreted as evidence that routine nursing documentation may contain contextual information related to patient acuity and clinical surveillance, rather than as evidence that nursing documentation or nursing activities directly influence patient outcomes.

The nursing care characteristics examined in this study should be interpreted as documentation-derived process indicators rather than direct therapeutic interventions, measures of nursing quality, or causal exposures. Measures such as nursing documentation density and monitoring frequency do not represent specific nursing actions intended to modify outcomes, but instead capture how patients are observed, assessed, and monitored within the clinical care environment. Several mechanisms may underlie the observed associations between these nursing care characteristics and in-hospital outcomes. First, higher documentation density and monitoring frequency are likely to reflect greater clinical complexity, as patients with more severe or unstable conditions require closer observation.^[20,36]^ Second, these characteristics may serve as markers of the clinical team’s real-time perception of risk, with increased nursing attention occurring in response to evolving or anticipated deterioration.^[19]^ Third, they may also indicate the intensity of monitoring resources available and deployed during the periprocedural period, particularly in higher-acuity care settings.^[22,37,38]^

In the context of acute myocardial infarction, where clinical status can change rapidly and the window for detecting complications is often narrow, more frequent nursing assessments and monitoring are commonly accompanied by heightened clinical vigilance.^[39–41]^ Accordingly, the associations observed in this study are more plausibly explained by the coupling of patient acuity, clinician concern, surveillance intensity, and care-setting complexity, rather than by a causal effect of nursing activities themselves. Reverse causation should also be considered: early clinical deterioration or adverse events may have prompted intensified monitoring and increased documentation. Therefore, documentation-derived nursing process indicators should be interpreted as contextual signals reflecting evolving clinical risk and surveillance intensity within the inpatient care process, rather than as direct evidence that nursing activities independently altered outcomes.

In this study, the addition of nursing care characteristics to a traditional clinical model resulted in a statistically significant improvement in discriminative performance, as reflected by increases in the area under the receiver operating characteristic curve. This finding suggests that nursing-related information may capture dimensions of in-hospital risk that are not fully represented by conventional demographic, clinical, and procedural variables alone. Importantly, the receiver operating characteristic analysis was conducted to evaluate discrimination – that is, the ability of a model to distinguish between patients who did and did not experience adverse in-hospital outcomes – rather than to develop or validate a predictive model. The observed improvement in AUC should therefore not be interpreted as evidence supporting the direct use of nursing care characteristics for individualized clinical decision-making or outcome prediction. Instead, these results highlight the potential value of nursing documentation as a complementary source of information within broader risk recognition frameworks.

Previous studies examining in-hospital outcomes among patients with acute myocardial infarction have predominantly focused on structural or organizational nursing indicators, such as ICU admission, levels of care, or staffing-related measures.^[23–26,42–45]^ While these indicators provide important contextual information, they offer limited insight into the dynamic processes of nursing observation and monitoring during hospitalization.^[27,28]^ The findings of the present study are broadly consistent with observational evidence emphasizing the importance of close monitoring and timely recognition in the management of critically ill patients. However, this study extends prior work by translating these concepts into quantifiable, process-oriented nursing care characteristics, such as documentation density and monitoring frequency, derived from routinely recorded clinical data.

The increase in AUC from 0.71 for the clinical model to 0.76 after adding selected nursing documentation indicators was statistically significant but clinically modest. The present study was not designed to replace these validated scores. Rather, it explored whether routinely documented nursing process indicators provide additional internal discriminative information beyond available clinical and PCI-related variables. In practical terms, an AUC increase of 0.05 suggests that nursing documentation variables may add some information about in-hospital risk heterogeneity, but this magnitude is insufficient to support direct clinical decision-making without calibration assessment, internal validation, external validation, and evaluation of clinical net benefit. Thus, these findings should be interpreted as hypothesis-generating evidence for nursing informatics and clinical surveillance research, not as validation of a bedside prediction tool.

These results have implications primarily for nursing informatics and hospital surveillance systems. Routinely collected nursing documentation may represent an underused data source for describing patient acuity, monitoring intensity, and evolving clinical complexity during hospitalization. Future informatics-based approaches could integrate structured nursing documentation with clinical, laboratory, electrocardiographic, and procedural data to support early-warning analytics or surveillance dashboards. However, such applications would require prospective testing, transparent algorithms, calibration, external validation, and careful assessment of workflow burden before implementation. The current findings do not support simply increasing the frequency of nursing documentation or monitoring for all patients. More documentation is not necessarily better care; documentation density may also be affected by documentation burden, staffing patterns, electronic record templates, institutional workflow, and local recording culture. These factors may distort documentation density and limit its portability across hospitals.

Subgroup and sensitivity analyses showed generally consistent directions of association across age, sex, AMI subtype, and care setting. However, these analyses should be interpreted cautiously because some subgroup-specific event counts were limited. The sensitivity analysis restricted to non-ICU patients and the analysis excluding early deaths supported the robustness of the main association between nursing documentation density and the primary composite outcome. Nevertheless, consistency across sensitivity analyses does not eliminate residual confounding, reverse causation, or surveillance bias. The results should therefore be regarded as internally supportive rather than externally confirmatory.

Several limitations should be acknowledged. First, this was a single-center retrospective observational study, which limits causal inference and generalizability. Although multivariable adjustment was performed, residual confounding from unmeasured disease severity, hemodynamic instability, workflow organization, staffing, and institutional care practices may remain. Second, nursing process indicators were derived from routine documentation and may be influenced by documentation habits, workload, electronic template design, staffing patterns, and local recording practices. Therefore, these indicators may not be directly comparable across hospitals with different nursing documentation systems. Third, although we attempted to reduce reverse causation by using a predefined early exposure window and excluding documentation after the 1st adverse event, this study could not fully determine whether nursing documentation preceded clinical deterioration or was generated in response to evolving complications. Fourth, documentation-derived variables may be affected by confounding by indication and clinical surveillance bias, particularly for rhythm monitoring, bleeding observation, fluid balance monitoring, and ICU-level care. Fifth, the model was assessed internally, and no external validation was performed; therefore, the findings should not be interpreted as establishing a validated risk prediction model. Sixth, the outcomes were limited to the in-hospital period, and no information on post-discharge or long-term prognosis was available. Finally, the findings should not be used to evaluate nursing performance or infer the effectiveness of specific nursing interventions.

## 5. Conclusion

In this single-center retrospective cohort of patients with acute myocardial infarction undergoing PCI, documentation-derived nursing process indicators were associated with in-hospital adverse outcomes and provided modest additional internal discriminative information beyond conventional clinical variables. These indicators should be interpreted as contextual markers of patient acuity, surveillance intensity, and care complexity, rather than as causal effects of nursing interventions or measures of nursing quality. Further prospective, multicenter studies with external validation are needed before these indicators can be incorporated into clinical surveillance or risk assessment systems.

## Acknowledgments

The authors sincerely thank all study participants for their invaluable contributions.

## Author contributions

**Conceptualization:** Haixia Ma, He Yin, Chunyan Wu, Jie Gao, Xiumei Yue, Hongbo Yu.

**Validation:** Chunyan Wu.

**Writing – original draft:** Hongbo Yu.

**Writing – review & editing:** Hongbo Yu.




















