# Supplementary material for: Documentation-derived nursing process indicators and in-hospital outcomes in patients with acute myocardial infarction undergoing PCI: A cohort study
Source: Medicine (Baltimore). 2026 Jun 19;105(25):e49375. doi: 10.1097/MD.0000000000049375 (PMC13286437; doi:10.1097/MD.0000000000049375)
Supplement: Supplementary file 1 [file medi-105-e49375-s001.docx]

**Supplementary Table S1. Reasons for exclusion from the final analytic cohort**

| **Screening step or exclusion reason** | **Number of patients** |
| --- | --- |
| Patients initially screened | 512 |
| Excluded after screening | 74 |
| Non-AMI diagnosis after adjudication | 21 |
| Did not undergo PCI during the index hospitalization | 15 |
| Incomplete or non-extractable nursing documentation | 18 |
| Missing in-hospital outcome data | 7 |
| Inter-hospital transfer or premature withdrawal from care | 9 |
| Severe non-cardiac comorbid conditions expected to dominate short-term prognosis | 4 |
| Final analytic cohort | 438 |

**Table note:**
Among the 18 patients excluded because of incomplete or non-extractable nursing documentation, 4 died early before sufficient nursing records were available for reliable exposure assessment. Exclusion categories were defined a priori based on routinely collected clinical and nursing records.
