# Supplementary material for: Documentation-derived nursing process indicators and in-hospital outcomes in patients with acute myocardial infarction undergoing PCI: A cohort study
Source: Medicine (Baltimore). 2026 Jun 19;105(25):e49375. doi: 10.1097/MD.0000000000049375 (PMC13286437; doi:10.1097/MD.0000000000049375)
Supplement: Supplementary file 2 [file medi-105-e49375-s002.docx]

**Supplementary Table S3. Coding rules for documentation-derived nursing process indicators**

| **Indicator** | **Data source** | **Included record types** | **Excluded entries** | **Coding format** | **Interpretation** |
| --- | --- | --- | --- | --- | --- |
| Total nursing assessment records | Electronic nursing documentation system | Admission nursing assessment, shift assessment, pre-PCI assessment, post-PCI assessment, symptom-focused assessment, complication-related nursing observation | Administrative entries, billing records, medication administration records, order acknowledgements, duplicate system-generated records | Continuous count | Cumulative number of structured clinical nursing assessment records |
| Vital sign monitoring records | Electronic nursing documentation system | Systolic and diastolic blood pressure, heart rate, respiratory rate, oxygen saturation, body temperature | Duplicate entries and automatically generated non-clinical system logs | Continuous count; records per exposure day | Frequency of documented physiological monitoring |
| Nursing documentation density | Electronic nursing documentation system | Nursing assessments, vital sign records, pain assessment records, bleeding/access-site observations, rhythm monitoring observations, fluid balance records, complication-related observations | Administrative entries, billing items, medication administration records, order acknowledgements, duplicate entries, non-clinical system logs | Continuous; eligible records per exposure day | Overall density of clinical nursing documentation during the predefined exposure window |
| Pain assessment documented | Electronic nursing documentation system | Structured pain score, symptom assessment, chest pain assessment, postprocedural pain observation | Non-clinical notes or duplicate entries | Binary: documented vs. not documented | Recorded pain-related nursing observation |
| Bleeding observation documented | Electronic nursing documentation system | Bleeding risk observation, access-site bleeding observation, hematoma observation, overt bleeding documentation | Generic administrative documentation without clinical observation | Binary: documented vs. not documented | Recorded bleeding or access-site surveillance; may reflect bleeding risk or active bleeding |
| Access-site inspection documented | Electronic nursing documentation system | Femoral or radial access-site inspection, puncture-site bleeding, hematoma, swelling, compression status | Routine administrative procedure logs | Binary: documented vs. not documented | Recorded access-site surveillance after PCI |
| Cardiac rhythm monitoring documented | Electronic nursing documentation system | Telemetry observation, rhythm monitoring record, arrhythmia-related nursing observation | Physician ECG reports without nursing documentation | Binary: documented vs. not documented | Recorded rhythm surveillance; may reflect telemetry indication or patient acuity |
| Fluid balance monitoring documented | Electronic nursing documentation system | Intake-output records, urine output monitoring, fluid balance charting | Medication administration logs without intake-output documentation | Binary: documented vs. not documented | Recorded fluid status surveillance; may reflect heart failure, renal dysfunction, shock, or ICU/CCU-level care |

**Table note:**
Documentation-derived nursing process indicators were extracted from routine nursing records and should be interpreted as process-oriented documentation markers rather than direct measures of nursing intervention, nursing quality, or causal exposure.
