# Supplementary material for: Documentation-derived nursing process indicators and in-hospital outcomes in patients with acute myocardial infarction undergoing PCI: A cohort study
Source: Medicine (Baltimore). 2026 Jun 19;105(25):e49375. doi: 10.1097/MD.0000000000049375 (PMC13286437; doi:10.1097/MD.0000000000049375)
Supplement: Supplementary file 3 [file medi-105-e49375-s003.docx]

**Supplementary Table S4. Validation of nursing documentation extraction**

| **Extracted variable** | **Validation sample size** | **Manual review positive, n** | **Extraction positive, n** | **Percent agreement, %** | **Cohen’s kappa** |
| --- | --- | --- | --- | --- | --- |
| Pain assessment documented | 120 | 101 | 100 | 96.7 | 0.87 |
| Bleeding observation documented | 120 | 91 | 90 | 95.8 | 0.86 |
| Access-site inspection documented | 120 | 94 | 95 | 95.0 | 0.84 |
| Cardiac rhythm monitoring documented | 120 | 79 | 80 | 96.7 | 0.93 |
| Fluid balance monitoring documented | 120 | 83 | 82 | 95.8 | 0.91 |
| Nursing documentation density | 120 | Not applicable | Not applicable | 94.2 | Not applicable |

**Table note:**
Structured nursing fields were electronically exported from the nursing information system. Semi-structured or free-text entries were manually reviewed according to prespecified coding rules. Percent agreement and Cohen’s kappa were used to evaluate extraction consistency for binary indicators. For nursing documentation density, validation was based on agreement between electronically exported eligible record counts and manual verification.
