# Supplementary material for: Documentation-derived nursing process indicators and in-hospital outcomes in patients with acute myocardial infarction undergoing PCI: A cohort study
Source: Medicine (Baltimore). 2026 Jun 19;105(25):e49375. doi: 10.1097/MD.0000000000049375 (PMC13286437; doi:10.1097/MD.0000000000049375)
Supplement: Supplementary file 4 [file medi-105-e49375-s004.docx]

**Supplementary Table S5. Missing data for variables included in the analysis**

| **Variable** | **Total N** | **Missing, n** | **Missing, %** | **Handling in analysis** |
| --- | --- | --- | --- | --- |
| Age | 438 | 0 | 0.0 | Complete |
| Sex | 438 | 0 | 0.0 | Complete |
| Body mass index | 438 | 0 | 0.0 | Complete |
| AMI subtype | 438 | 0 | 0.0 | Complete |
| Admission systolic blood pressure | 438 | 0 | 0.0 | Complete |
| Admission heart rate | 438 | 0 | 0.0 | Complete |
| Killip class | 438 | 0 | 0.0 | Complete |
| Left ventricular ejection fraction | 438 | 0 | 0.0 | Complete |
| Hypertension | 438 | 0 | 0.0 | Complete |
| Diabetes mellitus | 438 | 0 | 0.0 | Complete |
| Prior myocardial infarction | 438 | 0 | 0.0 | Complete |
| Current smoking | 438 | 0 | 0.0 | Complete |
| Serum creatinine | 438 | 0 | 0.0 | Complete |
| Peak troponin I | 438 | 0 | 0.0 | Complete; descriptive and sensitivity analysis |
| Number of diseased vessels | 438 | 0 | 0.0 | Complete |
| Infarct-related artery | 438 | 0 | 0.0 | Complete |
| Pre-PCI TIMI flow | 438 | 0 | 0.0 | Complete |
| Antiplatelet therapy | 438 | 0 | 0.0 | Complete |
| Anticoagulant therapy | 438 | 0 | 0.0 | Complete |
| Statin therapy | 438 | 0 | 0.0 | Complete |
| β-blocker use | 438 | 0 | 0.0 | Complete |
| ACEI/ARB/ARNI use | 438 | 0 | 0.0 | Complete |
| Total nursing assessment records | 438 | 0 | 0.0 | Complete |
| Vital sign monitoring records | 438 | 0 | 0.0 | Complete |
| Nursing documentation density | 438 | 0 | 0.0 | Complete |
| Pain assessment documented | 438 | 0 | 0.0 | Complete |
| Bleeding observation documented | 438 | 0 | 0.0 | Complete |
| Access-site inspection documented | 438 | 0 | 0.0 | Complete |
| Cardiac rhythm monitoring documented | 438 | 0 | 0.0 | Complete |
| Fluid balance monitoring documented | 438 | 0 | 0.0 | Complete |
| Primary composite endpoint | 438 | 0 | 0.0 | Complete |

**Table note:**
Only patients with complete and extractable nursing documentation and ascertainable in-hospital outcome data were included in the final analytic cohort. Therefore, variables included in the primary analysis had no missing values after application of the eligibility criteria.
