# Supplementary material for: Documentation-derived nursing process indicators and in-hospital outcomes in patients with acute myocardial infarction undergoing PCI: A cohort study
Source: Medicine (Baltimore). 2026 Jun 19;105(25):e49375. doi: 10.1097/MD.0000000000049375 (PMC13286437; doi:10.1097/MD.0000000000049375)
Supplement: Supplementary file 5 [file medi-105-e49375-s005.docx]

**Supplementary Table S6. Correlation matrix and variance inflation factors for nursing-related variables**

| **Variable** | **Total nursing assessment records** | **Vital sign monitoring frequency** | **Nursing documentation density** | **Cardiac rhythm monitoring** | **Fluid balance monitoring** | **ICU/CCU-level care** | **VIF** |
| --- | --- | --- | --- | --- | --- | --- | --- |
| Total nursing assessment records | 1.00 | 0.56 | 0.62 | 0.41 | 0.39 | 0.58 | 2.41 |
| Vital sign monitoring frequency | 0.56 | 1.00 | 0.58 | 0.44 | 0.46 | 0.61 | 2.18 |
| Nursing documentation density | 0.62 | 0.58 | 1.00 | 0.48 | 0.45 | 0.64 | 2.62 |
| Cardiac rhythm monitoring | 0.41 | 0.44 | 0.48 | 1.00 | 0.43 | 0.55 | 1.84 |
| Fluid balance monitoring | 0.39 | 0.46 | 0.45 | 0.43 | 1.00 | 0.52 | 1.92 |
| ICU/CCU-level care | 0.58 | 0.61 | 0.64 | 0.55 | 0.52 | 1.00 | 2.35 |

**Table note:**
Pearson or Spearman correlation coefficients were used for continuous variables, and phi coefficients or point-biserial correlations were used for binary-continuous combinations, as appropriate. Variance inflation factors were calculated from models including nursing-related variables and ICU/CCU-level care. No VIF exceeded 5, suggesting no severe multicollinearity.
