# Supplementary material for: Documentation-derived nursing process indicators and in-hospital outcomes in patients with acute myocardial infarction undergoing PCI: A cohort study
Source: Medicine (Baltimore). 2026 Jun 19;105(25):e49375. doi: 10.1097/MD.0000000000049375 (PMC13286437; doi:10.1097/MD.0000000000049375)
Supplement: Supplementary file 6 [file medi-105-e49375-s006.docx]

**Supplementary Table S10. Detailed discrimination, calibration, and internal validation of the clinical and extended models**

| **Model performance metric** | **Clinical model** | **Clinical + nursing documentation model** |
| --- | --- | --- |
| Apparent AUC | 0.71 | 0.76 |
| 95% CI for AUC | 0.66–0.76 | 0.71–0.81 |
| ΔAUC | Reference | 0.05 |
| DeLong P value | — | 0.012 |
| Optimism-corrected AUC | 0.70 | 0.75 |
| Brier score | 0.205 | 0.192 |
| Calibration intercept | 0.00 | 0.00 |
| Calibration slope | 0.94 | 0.96 |
| Hosmer–Lemeshow P value | 0.418 | 0.536 |

**Table note:**
The clinical model included age, sex, Killip class, hypertension, diabetes mellitus, prior myocardial infarction, left ventricular ejection fraction, serum creatinine, number of diseased vessels, infarct-related artery, and pre-PCI TIMI flow. The clinical + nursing documentation model additionally included nursing documentation density and documented cardiac rhythm monitoring. Internal validation was performed using bootstrap resampling. This analysis was intended to evaluate internal discrimination and calibration and was not designed to establish a validated clinical prediction tool.
