# Supplementary material for: Documentation-derived nursing process indicators and in-hospital outcomes in patients with acute myocardial infarction undergoing PCI: A cohort study
Source: Medicine (Baltimore). 2026 Jun 19;105(25):e49375. doi: 10.1097/MD.0000000000049375 (PMC13286437; doi:10.1097/MD.0000000000049375)
Supplement: Supplementary file 7 [file medi-105-e49375-s007.docx]

**Supplementary Table S7. Subgroup analyses for the association between nursing documentation density and the primary composite endpoint**

| **Subgroup** | **Category** | **N** | **Events, n** | **Adjusted OR** | **95% CI** | **P value** | **P for interaction** |
| --- | --- | --- | --- | --- | --- | --- | --- |
| Age | <65 years | 252 | 82 | 1.26 | 1.02–1.55 | 0.031 | 0.684 |
|  | ≥65 years | 186 | 86 | 1.33 | 1.06–1.68 | 0.014 |  |
| Sex | Male | 322 | 124 | 1.31 | 1.07–1.61 | 0.009 | 0.771 |
|  | Female | 116 | 44 | 1.24 | 1.01–1.55 | 0.046 |  |
| AMI subtype | STEMI | 276 | 121 | 1.32 | 1.07–1.63 | 0.010 | 0.638 |
|  | NSTEMI | 162 | 47 | 1.25 | 1.01–1.56 | 0.043 |  |
| Care setting | ICU/CCU-level care | 124 | 77 | 1.18 | 0.94–1.49 | 0.154 | 0.812 |
|  | No ICU/CCU-level care | 314 | 91 | 1.22 | 1.01–1.48 | 0.041 |  |

**Table note:**
Adjusted ORs were estimated for nursing documentation density per 1-record/day increase. Models were adjusted for age, sex, Killip class, hypertension, diabetes mellitus, prior myocardial infarction, left ventricular ejection fraction, serum creatinine, number of diseased vessels, infarct-related artery, and pre-PCI TIMI flow, except when the stratifying variable was part of the subgroup definition. Subgroup analyses were exploratory because some subgroup-specific event counts were limited.
