# Supplementary material for: Documentation-derived nursing process indicators and in-hospital outcomes in patients with acute myocardial infarction undergoing PCI: A cohort study
Source: Medicine (Baltimore). 2026 Jun 19;105(25):e49375. doi: 10.1097/MD.0000000000049375 (PMC13286437; doi:10.1097/MD.0000000000049375)
Supplement: Supplementary file 8 [file medi-105-e49375-s008.docx]

**Supplementary Table S8. Sensitivity analyses for the association between nursing documentation density and the primary composite endpoint**

| **Sensitivity analysis** | **Exposure definition or analytic restriction** | **Adjusted OR** | **95% CI** | **P value** |
| --- | --- | --- | --- | --- |
| Primary analysis | Nursing documentation density during the first 48 hours after admission, censored at first adverse event | 1.29 | 1.08–1.55 | 0.005 |
| Alternative early exposure window | Nursing documentation density during the first 24 hours after admission | 1.24 | 1.03–1.50 | 0.024 |
| PCI-based exposure window | Nursing documentation density during the first 24 hours after PCI | 1.21 | 1.01–1.46 | 0.039 |
| Whole-hospitalization exposure | Nursing documentation density across the entire hospitalization | 1.32 | 1.11–1.57 | 0.002 |
| Excluding early deaths | Patients with in-hospital death excluded | 1.25 | 1.04–1.51 | 0.018 |
| Non-ICU restriction | Analysis restricted to patients without ICU/CCU-level care | 1.22 | 1.01–1.48 | 0.041 |
| Alternative outcome definition | Primary composite excluding major bleeding | 1.27 | 1.06–1.52 | 0.010 |
| Additional adjustment | Model additionally adjusted for available admission systolic blood pressure and heart rate | 1.23 | 1.02–1.49 | 0.031 |
| Complete-case analysis | Complete-case dataset only | 1.28 | 1.07–1.54 | 0.007 |

**Table note:**
Adjusted ORs were estimated for nursing documentation density per 1-record/day increase. Primary models were adjusted for age, sex, Killip class, hypertension, diabetes mellitus, prior myocardial infarction, left ventricular ejection fraction, serum creatinine, number of diseased vessels, infarct-related artery, and pre-PCI TIMI flow. Sensitivity analyses were performed to evaluate the robustness of the main association across alternative exposure windows, alternative outcome definitions, exclusion of early deaths, restriction to non-ICU/CCU patients, and additional adjustment for available severity indicators.
