# Supplementary material for: Documentation-derived nursing process indicators and in-hospital outcomes in patients with acute myocardial infarction undergoing PCI: A cohort study
Source: Medicine (Baltimore). 2026 Jun 19;105(25):e49375. doi: 10.1097/MD.0000000000049375 (PMC13286437; doi:10.1097/MD.0000000000049375)
Supplement: Supplementary file 9 [file medi-105-e49375-s009.docx]

**Supplementary Table S2. Baseline clinical characteristics according to AMI subtype**

| **Characteristic** | **Missing, n (%)** | **Total (n = 438)** | **STEMI (n = 276)** | **NSTEMI (n = 162)** | **P value** |
| --- | --- | --- | --- | --- | --- |
| Age, years | 0 (0.0) | 61.42 ± 11.36 | 60.18 ± 11.02 | 63.48 ± 11.71 | 0.004 |
| Male sex, n (%) | 0 (0.0) | 322 (73.52) | 215 (77.90) | 107 (66.05) | 0.006 |
| Body mass index, kg/m² | 0 (0.0) | 25.18 ± 3.41 | 25.26 ± 3.36 | 25.05 ± 3.49 | 0.521 |
| Admission systolic blood pressure, mmHg | 0 (0.0) | 131.16 ± 21.56 | 129.48 ± 22.31 | 134.02 ± 19.91 | 0.034 |
| Admission heart rate, beats/min | 0 (0.0) | 80.84 ± 17.23 | 82.36 ± 17.88 | 78.25 ± 15.82 | 0.017 |
| Killip class ≥ II, n (%) | 0 (0.0) | 126 (28.77) | 96 (34.78) | 30 (18.52) | <0.001 |
| Left ventricular ejection fraction, % | 0 (0.0) | 52.36 ± 8.94 | 50.92 ± 8.76 | 54.82 ± 8.71 | <0.001 |
| Hypertension, n (%) | 0 (0.0) | 246 (56.16) | 149 (53.99) | 97 (59.88) | 0.232 |
| Diabetes mellitus, n (%) | 0 (0.0) | 158 (36.07) | 90 (32.61) | 68 (41.98) | 0.047 |
| Prior myocardial infarction, n (%) | 0 (0.0) | 52 (11.87) | 27 (9.78) | 25 (15.43) | 0.071 |
| Current smoking, n (%) | 0 (0.0) | 201 (45.89) | 139 (50.36) | 62 (38.27) | 0.014 |
| Serum creatinine, μmol/L | 0 (0.0) | 86.74 ± 21.83 | 84.92 ± 20.77 | 89.86 ± 23.24 | 0.028 |
| Peak troponin I, ng/mL | 0 (0.0) | 14.60 (7.20–27.80) | 23.50 (14.10–34.20) | 7.10 (3.80–11.90) | <0.001 |
| Number of diseased vessels ≥2, n (%) | 0 (0.0) | 231 (52.74) | 132 (47.83) | 99 (61.11) | 0.008 |
| Infarct-related artery, n (%) | 0 (0.0) |  |  |  | 0.012 |
| └ Left anterior descending artery | 0 (0.0) | 196 (44.75) | 138 (50.00) | 58 (35.80) |  |
| └ Right coronary artery | 0 (0.0) | 154 (35.16) | 92 (33.33) | 62 (38.27) |  |
| └ Left circumflex artery | 0 (0.0) | 88 (20.09) | 46 (16.67) | 42 (25.93) |  |
| Pre-PCI TIMI flow ≤1, n (%) | 0 (0.0) | 214 (48.86) | 173 (62.68) | 41 (25.31) | <0.001 |
| Antiplatelet therapy, n (%) | 0 (0.0) | 438 (100.00) | 276 (100.00) | 162 (100.00) | — |
| Anticoagulant therapy, n (%) | 0 (0.0) | 421 (96.12) | 267 (96.74) | 154 (95.06) | 0.386 |
| Statin therapy, n (%) | 0 (0.0) | 432 (98.63) | 273 (98.91) | 159 (98.15) | 0.520 |
| β-blocker use, n (%) | 0 (0.0) | 306 (69.86) | 189 (68.48) | 117 (72.22) | 0.410 |
| ACEI/ARB/ARNI use, n (%) | 0 (0.0) | 289 (65.98) | 177 (64.13) | 112 (69.14) | 0.286 |

**Table note:**
Values are presented as mean ± standard deviation, median (interquartile range), or number (percentage), as appropriate. P values compare STEMI and NSTEMI groups. Continuous variables were compared using Student’s t test or the Mann–Whitney U test, as appropriate. Categorical variables were compared using the χ² test or Fisher’s exact test, as appropriate.
