# Supplementary material for: Documentation-derived nursing process indicators and in-hospital outcomes in patients with acute myocardial infarction undergoing PCI: A cohort study
Source: Medicine (Baltimore). 2026 Jun 19;105(25):e49375. doi: 10.1097/MD.0000000000049375 (PMC13286437; doi:10.1097/MD.0000000000049375)
Supplement: Supplementary file 10 [file medi-105-e49375-s010.docx]

**Supplementary Table S9. Documentation-derived nursing process indicators stratified by clinical severity and care setting**

**Panel A. Stratification by Killip class**

| **Nursing process indicator** | **Killip I–II (n = 312)** | **Killip III–IV (n = 126)** | **P value** |
| --- | --- | --- | --- |
| Total nursing assessment records, n | 16.27 ± 5.84 | 23.91 ± 7.28 | <0.001 |
| Vital sign monitoring records per day, n | 8.41 ± 2.76 | 11.82 ± 3.14 | <0.001 |
| Pain assessment documented, n (%) | 253 (81.09) | 118 (93.65) | 0.001 |
| Bleeding observation documented, n (%) | 218 (69.87) | 110 (87.30) | <0.001 |
| Cardiac rhythm monitoring documented, n (%) | 178 (57.05) | 108 (85.71) | <0.001 |
| Nursing documentation density, records/day | 4.11 ± 1.32 | 5.89 ± 1.61 | <0.001 |

**Panel B. Stratification by periprocedural complications**

| **Nursing process indicator** | **No complications (n = 331)** | **≥1 complication (n = 107)** | **P value** |
| --- | --- | --- | --- |
| Total nursing assessment records, n | 17.02 ± 6.01 | 22.81 ± 7.46 | <0.001 |
| Vital sign monitoring records per day, n | 8.72 ± 2.91 | 11.26 ± 3.37 | <0.001 |
| Pain assessment documented, n (%) | 271 (81.87) | 100 (93.46) | 0.002 |
| Bleeding observation documented, n (%) | 236 (71.30) | 92 (85.98) | 0.003 |
| Cardiac rhythm monitoring documented, n (%) | 189 (57.10) | 97 (90.65) | <0.001 |
| Nursing documentation density, records/day | 4.29 ± 1.41 | 5.96 ± 1.68 | <0.001 |

**Panel C. Stratification by ICU/CCU-level care**

| **Nursing process indicator** | **No ICU/CCU-level care (n = 314)** | **Any ICU/CCU-level care (n = 124)** | **P value** |
| --- | --- | --- | --- |
| Total nursing assessment records, n | 15.09 ± 4.82 | 26.58 ± 7.94 | <0.001 |
| Vital sign monitoring records per day, n | 7.62 ± 2.41 | 13.74 ± 3.89 | <0.001 |
| Pain assessment documented, n (%) | 255 (81.21) | 116 (93.55) | 0.002 |
| Bleeding observation documented, n (%) | 219 (69.75) | 109 (87.90) | <0.001 |
| Cardiac rhythm monitoring documented, n (%) | 168 (53.50) | 118 (95.16) | <0.001 |
| Nursing documentation density, records/day | 3.93 ± 1.12 | 6.38 ± 1.71 | <0.001 |

**Table note:**
Values are presented as mean ± standard deviation or number (percentage), as appropriate. ICU/CCU-level care was defined as any intensive or coronary care unit-level care during hospitalization, including direct ICU/CCU admission at presentation or immediately after PCI and subsequent ICU transfer after initial non-ICU management. P values were calculated using Student’s t test or the Mann–Whitney U test for continuous variables and the χ² test or Fisher’s exact test for categorical variables, as appropriate.
